# One Size Does Not Fit All: The Case for Translational Medicine

**DOI:** 10.5041/RMMJ.10364

**Published:** 2019-04-18

**Authors:** Yehuda Chowers

**Affiliations:** 1 Department of Gastroenterology, Rambam Health Care Campus, Haifa, Israel; 2 The Ruth & Bruce Rappaport Faculty of Medicine, Technion–Israel Institute of Technology, Haifa, Israel

**Keywords:** Crohn’s disease, inflammatory bowel disease, personalized medicine, systems immunology, ulcerative colitis

## Abstract

Therapy for inflammatory bowel diseases (IBD) has developed during recent years. Despite the availability of new therapeutic modalities, overall therapy success remains modest, and complete remission is usually achieved and maintained in approximately 30% of patients only. This observation can be explained by a number of reasons. First, the involvement of multiple genetic loci combined with differential environmental exposures suggests that IBD represent a continuum of disorders rather than distinct homogeneous disease entities. This diversity is translated into different disease course patterns, wherein some patients experience quiescent disease whereas others suffer from a relentless disease course. Hence, basic disease pathogenesis sets the stage for differential treatment responses. To date, IBD therapy is based on immunosuppression which does not take basic disease variability into account. Treatments are prescribed based on statistical considerations related to the response of the average patient in clinical trials rather than on personal considerations. Treatment outcomes can potentially be improved if physiologic considerations are integrated into the drug selection process. In one approach, drugs can be targeted at known patient dysfunctional processes such as in the case of patients carrying autophagy-related genetic polymorphisms being treated with rapamycin, a drug that inhibits mTOR inhibitor and enhances autophagy. Another alternative would be to use a systems approach to perform unsupervised, high-throughput screening in order to derive predictive treatment biomarkers and mechanistic insights associated with response to specific drug therapy. Additional predictive markers for drug safety are needed as well. Caveats and directions for needed future studies are outlined.

## INTRODUCTION

Inflammatory bowel diseases (IBD) are traditionally classified into Crohn’s disease (CD) and ulcerative colitis (UC). The etiology and pathogenesis of CD and UC are still elusive despite multiple studies aimed at increasing our understanding of these issues. However, a generally accepted concept is that tissue damage is caused by immune activation, and hence both diseases are considered to originate from immune system dysfunction. Because of this ambiguity regarding the sequence of events which leads to the tissue-damaging immune activation, therapy is non-specific and is focused on suppressing immune activity using an array of agents, differing in their mechanism of action and targets. Therefore, it is not surprising—but still disappointing—that uniformly there are relatively poor outcomes achieved by using these diverse therapeutic modalities, regardless of whether small molecules or monoclonal antibodies are used, or whether the target is a specific molecule, or a basic cellular mechanism that affects many inflammatory pathways.

## ISSUES THAT COMPLICATE THERAPEUTIC DECISION-MAKING

Anti-TNF antibodies have revolutionized IBD care and are indicated for the treatment of inflammatory and penetrating forms of CD. However, in fact these drugs exhibit relatively disappointing outcomes. In the major clinical trials, infliximab administered at the standard 5 mg/kg dosage has demonstrated an initial 58.4% of overall response two weeks post infusion. Responding patients continued to a maintenance trial in which only 39% were in remission at week 30, yielding only 22.8% patients in remission out of the intention-to-treat population.^1^ Similar results were obtained with other anti-TNF agents such as adalimumab^2^ and certolizumab^3^ and in UC trials as well.^4^ These relatively poor response rates were demonstrated with other agents targeting different pathways such as the anti-migration agent vedolizumab^5^ and the recently approved anti-P40 antibody ustekinumab.^6^ As noted, this range of response rates is not limited to monoclonal antibodies and was documented for small molecules such as thiopurines,^7^ methotrexate,^8^ and the more recently develop JAK inhibitor tofacitinib^9^ approved for UC.

Therapeutic decisions are further complicated by the fact that immune suppression is associated with potential severe side effects including malignancies^10^ and both serious and opportunistic infections.^11^ These complications, which may occur without imparting a therapeutic advantage on the patient, necessitate careful and precise choice of therapy. Therefore, a careful balance of medical cost–benefit should be made before therapy selection.

Ideally, this type of therapy should be prescribed either to patients who are symptomatic, or to those in whom disease is predicted to progress and cause significant tissue damage and functional loss. Furthermore, these therapies should be restricted only to patients who have a high chance to respond, preferably with a low risk for side effects. Clearly, this goal has not been achieved.

What can be the reasons for this relatively poor drug performance? An in-depth look into the complex etiology of IBD and the difficulty to predict disease course may offer some insights into this problem.

## COMPLEXITIES OF IBD AND DISEASE COURSE PREDICTION

Despite the common division of IBD into CD and UC, this in fact is probably an oversimplification, and IBD may represent a syndrome with different etiologic and pathogenic factors which are differentially responsive to diverse modes of therapy. For example, in a recent study, which is the largest of its type, the genetic background of 29,838 IBD patients (16,902 with CD, 12,597 with UC) was evaluated using immunochip arrays.^12^ The authors demonstrated that anatomic distribution did not change over time, whereas disease phenotype (stricturing and penetrating complications) changed, suggesting a more significant effect of genetics on the anatomic distribution rather than on complication occurrence and a possible environmental effect on the latter. Furthermore, anatomical distribution was better explained by genetic risk scores than by single genetic variants, pointing at the complex genetic background of IBD. Finally, the authors suggested that IBD represent a continuum of disorders rather than specific disease entities. This observation concurs with the results of IBD genetic association studies in which 163 disease-associated loci were found. Of note, 110 of the 163 were associated with both CD and UC, 30 were specific for CD, and 23 were specific for UC.^13^ Furthermore, a recent study has demonstrated that even when a single organ is involved, transcriptomic, DNA accessibility, and pathway enrichment analysis revealed two distinct phenotypes both in the colon and in the ileum.^14^ This observation suggests that an attempt to accurately categorize IBD may necessitate analysis of functional modes within the involved organ which is the therapeutic target, in order to tailor effective therapy. To date, drug labels define indications aimed at CD or UC and do not take into account the extreme variability of these disorders.

Disease variability and environmental exposures are clinically translated into disease course. Indeed, variability in disease course and outcomes has been well documented,^15^ and demonstrated that both CD and UC patients may run different disease courses, with some having an indolent uneventful course, whereas others have a severe course characterized by continuous disease activity. Clearly, an ability to predict this course would be of importance, since one would prefer to avoid an aggressive therapeutic regimen in patients who are not at risk for future disease activity and organ damage. Nonetheless, such predictive factors are not readily available, and those that were investigated vary according to selected outcomes. For example, Beaugerie and colleagues used a single-center observational cohort and showed that disease onset at age <40, perianal disease, and need for steroid treatment predict a disabling disease course.^16^ A complicated course was defined as more than two steroid courses, or steroid dependence, hospitalization for flare-up or disease complication, presence of extra-intestinal complications, disabling symptoms, need for immunosuppressive therapy, and need for surgery. Using the same definitions, Loly et al.^17^ found a lower percentage of disabling disease (60%) in their cohort compared to that of the Beaugerie study (85.2%). They also found perianal lesions at diagnosis and early steroid use as a risk for disabling disease. However, they found that weight loss and stricturing disease at diagnosis were associated with a risk for multiple small-bowel resections, colonic resection, stoma, or complex perianal disease. These differences show how difficult it may be to form inclusive definitions based on clinical retrospective data. The authors suggested that the differences may stem from different referral practices in the two centers. Moreover, as noted, use of immunosuppressing agents was part of the definition for disabling disease. Since immunosuppressives are used to treat and prevent the other outcomes defined as disabling disease, this criterion may not represent by itself a bona fide disabling disease course. These observations raise a question related to the reproducibility of tools used for predicting disease course based solely on clinical variables.

Further insight may be gained by using translational tools that probe factors associated with immune activity, which is the physiologic system actually inflicting tissue damage. An example of the potential of this approach is the study by Lee and colleagues.^18^ These investigators found a peripheral blood transcriptional signature in CD8 T cells which predicted a worse disease course. By comparison, this mode was superior to previously proposed serology and clinical factor combinations. Regrettably, despite its impressive results, similar studies were not performed. Thus, this part of the equation is still unresolved as well.

Besides adjustment of therapy according to the projected disease course and side effects, a key component for selection of a specific drug is its chance for a beneficial therapeutic success. To date, the ultimate supporting evidence for drug selection is in the results of double-blind placebo controlled trials. However, despite the fact that this methodology is indeed the most powerful and objective tool to judge drug efficacy, its applicability for the individual patient is questionable because it relies on the patient’s statistical chance of being part of the responding patient population, which in the case of IBD will allow approximately 30% or less to achieve remission. Can these poor outcomes be improved?

## ALTERNATIVE APPROACHES FOR THERAPY SELECTION

A number of alternative approaches for therapy selection can be supported by existing evidence. The first would be to match known physiologic defects with an appropriate drug effect. An example of such putative combination is the case of autophagy and CD. Genetic studies have demonstrated a linkage between polymorphisms in autophagy-associated genes and CD.^19^ These were coupled with functional studies suggesting a role in inflammation.^20^ Rapamycin is a drug which inhibits mTOR, a negative regulator of the autophagy pathway, resulting in its activation.^21^ Indeed, a case report and a case series described successful treatment of CD patients with rapamycin.^22^ However, a controlled trial failed to show its efficacy.^23^ No genetic testing was performed in this trial, and thus the potential for mechanism-driven therapy was actually not tested. Another example of potential targeted therapy is the use of thiopurines. These agents were described to have a number of mechanisms of action. Recently, they were shown to enhance innate immune function by inhibiting p21Rac1, leading to enhanced bacterial killing, IL-8 secretion, and phagocytosis.^24^ This effect may correct the implied immune deficiency state suggested to be associated with the most prevalent genetic polymorphism in NOD2 in the Caucasian population.^25^ In addition, thiopurines were also described to induce autophagy in epithelial cells,^26^ providing a second defined therapeutic target. Finally, thiopurines were shown to induce apoptosis of proliferating cells,^27^ thereby explaining their ability to prevent immunogenicity even following primary therapeutic failure.^28^ Nonetheless, no trials applying mechanism-based genetic testing were performed, a fact that may contribute to the insufficient response rates observed with thiopurines similar to other drugs used in IBD therapy.^7^

A second approach may involve a systematic screening for biomarkers associated with response to treatment. The search for biomarkers of the response to anti-TNF agents provides examples of both the problems and the hopes associated with this approach. A pioneering experiment was performed by Arijs and colleagues who performed gene expression studies in infliximab-treated patients.^29^ These investigators identified 212 gene sets which were differentially expressed between responders and non-responders. More recently, an additional study used gene expression data and identified pretreatment mucosal oncostatin M (OSM) expression as a marker for non-response to anti-TNF therapy with an AUC of 95%.^30^ In parallel, we have used meta-analysis of public domain data (including data from Arijs et al.) and correction for cell proportions, which normalizes the gene expression data according to the cellular composition (deconvolution),^31^ and found that prevalence of high pretreatment mucosal plasma cells and inflammatory macrophages predicted non-response to infliximab. We further found that upregulation of mucosal triggering receptor expressed on myeloid cells 1 (TREM-1) expression was associated with non-response to anti-TNF therapy, whereas its increased expression in the peripheral blood was associated with response with an AUC of 94%.^32^ These findings, however, provoked a follow-up publication by another group in which peripheral blood TREM-1 expression was found to predict non-response.^33^

What could be the reasons for these contradictory results using similar analytic methods (gene expression)? First, the definitions for response appear to be highly significant. Thus, whereas our group^32^ used a clinical algorithm to determine response, mucosal healing was used as an endpoint in the other publication.^33^ Furthermore, whereas both publications^32^,^33^ used drug levels and anti-drug antibody measurements to define drug-related responses, no such definitions were used in the publications by Arijs et al. and West et al.^29^,^30^ Thus, outcome definitions have a marked effect on the end results of such studies. Second, analytic methodologies may markedly affect the results. Because absolute gene expression depends on the content of tissue-infiltrating cells, wherein transcripts derived from high numbers of infiltrating cells will dominate the findings, adjusting for cell proportions can potentially completely change the end result. This is well demonstrated when comparing the end result of Arijs et al.^29^ to our recent publication in which adjustment to cell proportions was used. Therefore, agreed outcomes and standardized analytic methodologies applied to different cohorts may pave the way to insightful results that will necessitate solid prospective clinical confirmation. Notwithstanding these discrepancies, in addition to potential predictive therapeutic biomarkers, such studies yield important insights into novel inflammatory pathways which may be of importance for development of future therapeutic agents that can be effective in todays’ refractory (non-responding) patient populations.

## LOOKING TO THE FUTURE

Ongoing studies verifying the results by Lee and colleagues^18^ hold promise for further validation of more accurate means of predicting disease course, as well as additional investigations that address disease follow-up and flare prediction by meticulous intestinal imaging using magnetic resonance enterography (MRE) and capsule endoscopy.^34^ In addition, drug response prediction might be further improved by adding bacterial and metabolic factors to the overall systems approach.^35^,^36^

Thus, by combining multimodal analysis—spanning natural history and treatment response biomarkers—and analysis of biologic interactions before and during treatment, we may be able to generate improved and timely treatment schemes.

## CONCLUSION

In conclusion, current evidence-based approaches using drugs that have proved to be efficacious in a limited proportion of patients in selected cohorts, followed by non-specific application to common clinical patient cohorts, yield poor outcomes. Therapeutic results can be significantly improved if individual patient characteristics are considered and carefully evaluated by mechanistic workup. Further studies are needed to refine the proper outcome measures and analytic methodologies in order to obtain validated results that can be used in clinical practice.

## Abbreviations

CD: Crohn’s disease
IBD: inflammatory bowel diseases
UC: ulcerative colitis.

## References

[1] Hanauer SB, Feagan BG, Lichtenstein GR (2002). Maintenance infliximab for Crohn’s disease: the ACCENT I randomised trial. Lancet.

[2] Colombel J, Sandborn WJ, Rutgeerts P (2007). Adalimumab for maintenance of clinical response and remission in patients with Crohn’s Disease: the CHARM Trial. Gastroenterology.

[3] Schreiber S, Khaliq-Kareemi M, Lawrance IC (2007). Maintenance therapy with certolizumab pegol for Crohn’s disease. N Engl J Med.

[4] Rutgeerts P, Sandborn WJ, Feagan BG (2005). Infliximab for induction and maintenance therapy for ulcerative colitis. N Engl J Med.

[5] Sandborn WJ, Feagan BG, Rutgeerts P (2013). Vedolizumab as induction and maintenance therapy for Crohn’s disease. N Engl J Med.

[6] Feagan BG, Sandborn WJ, Gasink C (2016). Ustekinumab as induction and maintenance therapy for Crohn’s disease. N Engl J Med.

[7] Ruffolo C, Scarpa M, Bassi N (2010). Infliximab, azathioprine, or combination therapy for Crohn’s disease. N Engl J Med.

[8] Feagan BG, Fedorak RN, Irvine EJ (2000). A comparison of methotrexate with placebo for the maintenance of remission in Crohn’s disease. N Engl J Med.

[9] Sandborn WJ, Su C, Sands BE (2017). Tofacitinib as induction and maintenance therapy for ulcerative colitis. N Engl J Med.

[10] Lemaitre M, Kirchgesner J, Rudnichi A (2017). Association between use of thiopurines or tumor necrosis factor antagonists alone or in combination and risk of lymphoma in patients with inflammatory bowel disease. JAMA.

[11] Kirchgesner J, Lemaitre M, Carrat F (2018). Risk of serious and opportunistic infections associated with treatment of inflammatory bowel diseases. Gastroenterology.

[12] Cleynen I, Boucher G, Jostins L (2016). Inherited determinants of Crohn’s disease and ulcerative colitis phenotypes: a genetic association study. Lancet.

[13] Cleynen I, Vermeire S (2015). The genetic architecture of inflammatory bowel disease. Curr Opin Gastroenterol.

[14] Weiser M, Simon JM, Kochar B (2018). Molecular classification of Crohn’s disease reveals two clinically relevant subtypes. Gut.

[15] Solberg IC, Vatn MH, Høie O (2007). Clinical course in Crohn’s disease: results of a Norwegian population-based ten-year follow-up study. Clin Gastroenterol Hepatol.

[16] Beaugerie L, Seksik P, Nion-Larmurier I (2006). Predictors of Crohn’s disease. Gastroenterology.

[17] Loly C, Belaiche J, Louis E (2008). Predictors of severe Crohn’s disease. Scand J Gastroenterol.

[18] Lee JC, Lyons PA, McKinney EF (2011). Gene expression profiling of CD8+ T cells predicts prognosis in patients with Crohn disease and ulcerative colitis. J Clin Invest.

[19] Hampe J, Franke A, Rosenstiel P (2007). A genome-wide association scan of nonsynonymous SNPs identifies a susceptibility variant for Crohn disease in ATG16L1. Nat Genet.

[20] Hoefkens E, Nys K, John JM (2013). Genetic association and functional role of Crohn disease risk alleles involved in microbial sensing, autophagy, and endoplasmic reticulum (ER) stress. Autophagy.

[21] Tsang CK, Qi H, Liu LF (2007). Targeting mammalian target of rapamycin (mTOR) for health and diseases. Drug Discov Today.

[22] Massey DCO, Bredin F, Parkes M (2008). Use of sirolimus (rapamycin) to treat refractory Crohn’s disease. Gut.

[23] Reinisch W, Panés J, Lémann M (2008). A multicenter, randomized, double-blind trial of everolimus versus azathioprine and placebo to maintain steroid-induced remission in patients with moderate-to-severe active Crohn’s disease. Am J Gastroenterol.

[24] Parikh K, Zhou L, Somasundaram R (2014). Suppression of p21Rac signaling and increased innate immunity mediate remission in Crohn’s disease. Sci Transl Med.

[25] van Heel DA, Ghosh S, Butler M (2005). Muramyl dipeptide and toll-like receptor sensitivity in NOD2-associated Crohn’s disease. Lancet.

[26] Oancea I, Movva R, Das I (2017). Colonic microbiota can promote rapid local improvement of murine colitis by thioguanine independently of T lymphocytes and host metabolism. Gut.

[27] Ben-Horin S, Goldstein I, Fudim E (2009). Early preservation of effector functions followed by eventual T cell memory depletion: a model for the delayed onset of the effect of thiopurines. Gut.

[28] Bar-Yoseph H, Waterman M, Almog R (2017). Prevention of antidrug antibody formation to infliximab in Crohn’s patients with prior failure of thiopurines. Clin Gastroenterol Hepatol.

[29] Arijs I, Li K, Toedter G (2009). Mucosal gene signatures to predict response to infliximab in patients with ulcerative colitis. Gut.

[30] West NR, Hegazy AN, Owens BMJ (2017). Oncostatin M drives intestinal inflammation and predicts response to tumor necrosis factor–neutralizing therapy in patients with inflammatory bowel disease. Nat Med.

[31] Shen-Orr SS, Tibshirani R, Khatri P (2010). Cell type–specific gene expression differences in complex tissues. Nat Methods.

[32] Gaujoux R, Starosvetsky E, Maimon N (2018). Cell-centred meta-analysis reveals baseline predictors of anti-TNFα non-response in biopsy and blood of patients with IBD. Gut.

[33] Verstockt B, Verstockt S, Blevi H (2018). TREM-1, the ideal predictive biomarker for endoscopic healing in anti-TNF-treated Crohn’s disease patients?. Gut.

[34] Ben-Horin S, Lahat A, Amitai MM (2018). 599 - Comprehensive video capsule endoscopy-based monitoring predicts short and long-term risk of disease flares in small bowel Crohn’s disease: a prospective cohort study. Gastroenterology.

[35] Ananthakrishnan AN, Luo C, Yajnik V (2017). Gut microbiome function predicts response to anti-integrin biologic therapy in inflammatory bowel diseases. Cell Host Microbe.

[36] Zhou Y, Xu ZZ, He Y (2018). Gut microbiota offers universal biomarkers across ethnicity in inflammatory bowel disease diagnosis and infliximab response prediction. mSystems.

